# Cytoprotective Effect of American Ginseng in a Rat Ethanol Gastric Ulcer Model

**DOI:** 10.3390/molecules19010316

**Published:** 2013-12-27

**Authors:** Chi-Chang Huang, Yi-Ming Chen, Dean-Chuan Wang, Chien-Chao Chiu, Wan-Teng Lin, Chih-Yang Huang, Mei-Chich Hsu

**Affiliations:** 1 Graduate Institute of Sports Science, National Taiwan Sport University, Taoyuan 33301, Taiwan; 2 Department of Sports Medicine, Kaohsiung Medical University, Kaohsiung 80708, Taiwan; 3 Department of Hospitality, Tunghai University, Taichung 40704, Taiwan; 4 Graduate Institute of Basic Medical Science, China Medical University, Taichung 40402, Taiwan; 5 Department of Health and Nutrition Biotechnology, Asia University, Taichung 41354, Taiwan

**Keywords:** American ginseng, gastric ulcer, inflammation, apoptosis

## Abstract

*Panax quinquefolium L*. (American Ginseng, AG) is one of the most popular herbal medicines in the World. We aimed to investigate whether chronic (28-day) supplementation with AG could protect against ethanol-induced ulcer in gastric tissue. Furthermore, we investigated the possible molecular mechanisms leading to AG-mediated gastric mucosal protection. We randomized 32 male Wistar rats into four groups for treatment (n = 8 per group): supplementation with water (vehicle) and low-dose (AG-1X), medium-dose (AG-2X) and high-dose (AG-5X) AG at 0, 250, 500, and 1250 mg/kg, respectively. In the first experiment, animals were fed vehicle or AG treatments for 4 weeks. At day 29, 75% ethanol was given orally to each animal at 10 mL/kg to induce gastric ulceration for 2 h. In a second experiment, animals were pretreated orally with each treatment for 1 hr before a single oral administration of ethanol (70%, 10 mL/kg). Trend analysis revealed that AG treatments inhibited ethanol-induced gastric mucosal damage. AG supplementation dose-dependently decreased the pro-inflammatory levels of interleukin 1β and cyclooxygenase 2 and the expression of pro-apoptotic proteins tBid, cytochrome C, and caspases-9 and -3 and increased the levels of anti-apoptotic proteins Bcl-2, Bcl-xL and p-Bad. AG could have pharmacological potential for treating gastric ulcer.

## 1. Introduction

Dietary supplements or complementary alternative medicines are popular self-administered remedies for gastric ulcer. * Panax quinquefolium L*. (American ginseng) has long been cultivated widely in North America and widely used in Eastern medicine and as a health supplement. American ginseng has potential as a chemopreventive agent [1], improves cancer-related fatigue [2], has immunomodulation [3] and anti-stress properties [4] and improves hyperglycemia [5].

The functional ingredients of ginseng include ginsenosides, polysaccharides, peptides, polyacetylenic alcohols, and fatty acids [6,7]. The main functional ingredients are thought to be the ginsenosides [8]. The ginsenosides content of American ginseng is located in the following decreasing order: leaf > root-hair > rhizome > root > stem [9]. In general, the ginsenosides Rb1, Re, Rd, Rc, Rg1 and Rb3, the six major saponins, account for more than 70% of the total ginsenoside content in American ginseng [10]. However, to our knowledge, no studies have investigated the mechanism of the anti-ulcer potential of gingseng. The present study thus investigated the protective effect of American ginseng extract in experimental models of gastric ulceration in rats. We further examined the possible mechanism of this effect.

## 2. Results and Discussion

### 2.1. Body Weight

**Figure 1:**
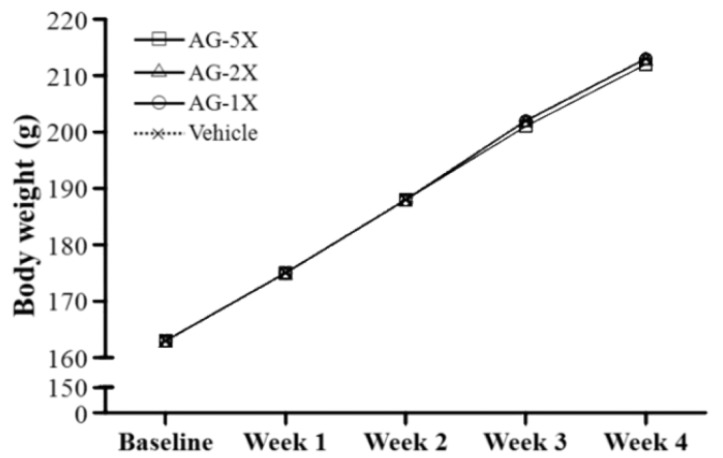
The changes of body weight of rats supplemented with vehicle, 250, 500 and 1250 mg/kg AG (AG-1X, AG-2X and AG-5X) for 28 days. Data are mean ± SEM for n = 8 rats per group.

The initial body weight at the beginning of the study for the different groups was 162.6 ± 4.7, 162.8 ± 4.6, 163.1 ± 4.7 and 163.1 ± 4.6 g, respectively, with no significant difference between groups (Figure 1).

After 28-day AG supplementation, the final body weight at the end of the study was 213.1 ± 6.0, 212.8 ± 4.6, 212.5 ± 4.1 and 211.9 ± 3.8 g, respectively, with no significant difference between groups. Significant changes in body and organ weight are known to result from oral toxicity or oxidative stress-induced organ damage. Previous studies indicated no significant alterations in body and tissue weight with AG administration over 15 consecutive days [11]. Therefore, 1250-mg/kg AG may not have toxic effects.

### 2.2. Effect of AG Supplementation on Ethanol-induced Gastric Mucosal Damage

**Figure 2:**
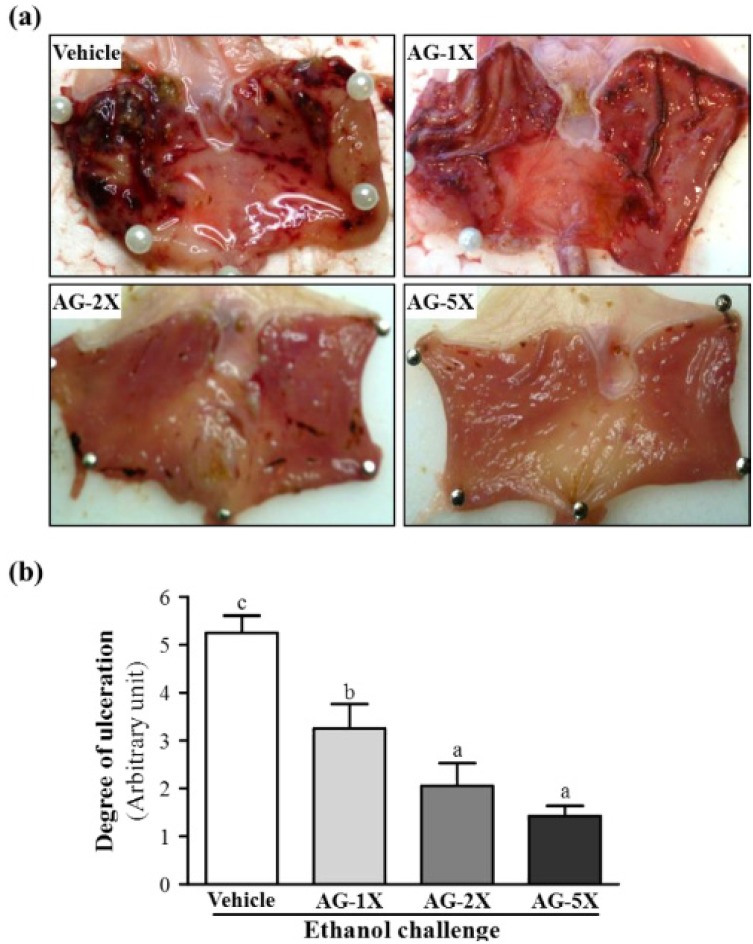
Gross evaluation of rat stomachs with AG treatment. (**a**) Gastric mucosa with pre-treatment with water (vehicle), low-dose (AG-1X), medium-dose (AG-2X) and high-dose (AG-5X) AG at 0, 250, 500 and 1250 mg/kg, respectively, for 4 weeks. At day 29, 75% ethanol was given orally to each animal at 10 mL/kg to induce gastric ulceration for 2 h; and (**b**) The degree of ulceration was expressed by a lesion score of 0 to 6. Data are mean ± SEM. Different letters (a, b, c) indicate significant difference at *p* < 0.05.

The gross appearance of rat stomachs treated with 75% ethanol to induce gastric mucosal damage is in Figure 2a. We evaluated whether AG treatment could protect against ethanol-induced mucosal damage in rat stomachs by assigning a pathology score. Ethanol-induced severe damage to the gastric mucosa was shown as elongated bands of haemorrhage in the vehicle group. 

This finding was similar to previous reports showing that ethanol intoxication caused extensive and acute haemorrhagic lesions on the mucosal surface of the gastric tissue and damaged gastric layers [12]. AG-2X and AG-5X produced an almost normal appearance of intact stomachs. Grading of lesions was 5.25 ± 0.36, 3.25 ± 0.5, 2.06 ± 0.47 and 1.43 ± 0.21 for the vehicle, AG-250, AG-500, and AG-1250 groups, respectively, which was significantly decreased by 38.1% (*p* < 0.005), 60.8% (*p* < 0.001) and 72.8% (*p* < 0.001), respectively, as compared with vehicle treatment (Figure 2b). In the trend analysis, gross lesions were dose-dependently decreased (*p* < 0.001). These pathological changes were almost completely ameliorated with 28-day AG supplementation (Figure 2b). Therefore, American ginseng may have dose-dependent gastro-protective activity.

### 2.3. Histology

**Figure 3:**
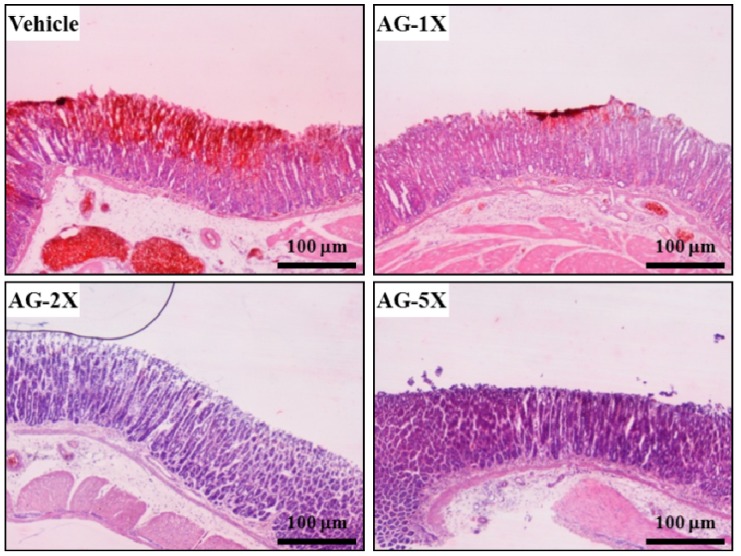
Histology of rat gastric mucosa with AG treatment. Rats were treated as in Figure 2. (H&E stain, magnification: ×40, Scale bar, 100 μm).

Ethanol-induced gastric ulcers are due to many mechanisms, including depletion of gastric mucus and impaired mucosal permeability, and leads to increased leakage of hydrogen ions from the lumen and decreased transluminal membrane potential difference [13]. Our vehicle-treated rats showed significant and extensive damage in the gastric mucosa, with edema in the submucosal layer (Figure 3). Pre-treatment with AG at 250, 500 and 1250 mg/kg, gave relatively better protection, as observed by decreased ulcer area, reduced or complete absence of edema, and flattening of the mucosal fold. Histology studies confirmed the efficacy of AG supplementation in preventing ethanol-induce hemorrhage and necrosis in the superficial layer of the gastric mucosa.

### 2.4. Effect of AG Supplementation on Ethanol-induced Expression of Inflammation-related Proteins in Rat Stomachs

**Figure 4:**
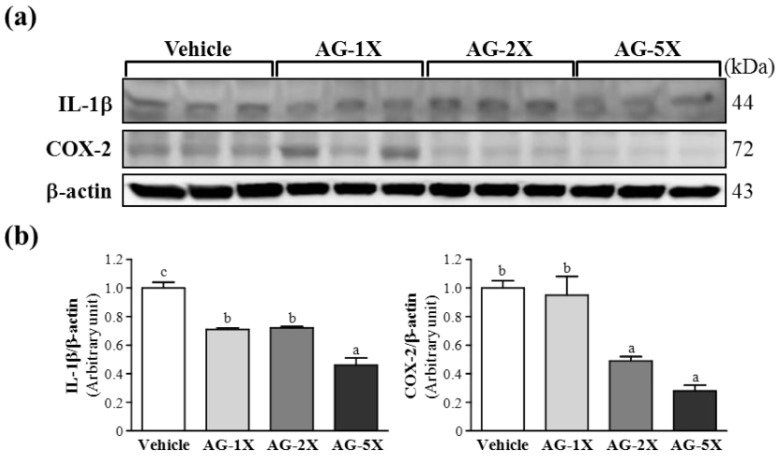
Effect of AG supplementation on ethanol-induced expression of inflammatory proteins in rat stomachs. Rats were pretreated with 0 (Vehicle), 250 (AG-1X), 500 (AG-2X) and 1250 (AG-5X) mg/kg AG for 1 h before a single oral administration of ethanol (70%, 10 mL/kg). At 4 h after ethanol administration, animals were killed and stomachs were collected. (**a**) Western blot analysis and quantification of protein levels of IL-1β and COX-2; and (**b**) Densitometry analysis was performed to quantify the expression levels of detected proteins. Data are mean ± SEM (n = 8 mice per group). Different letters (a, b, c) indicate a significant difference at *p* < 0.05.

We examined the protein levels of interleukin 1β (IL-1β) and cyclooxygenase 2 (COX-2) in ethanol-treated gastric tissues. IL-1β protein levels were significantly decreased by 29.3% (*p* = 0.0003), 27.5% (*p* = 0.0004) and 54.4% (*p* < 0.0001) with AG-1X, AG-2X and AG-5X treatment, respectively, as compared with vehicle treatment (Figure 4). 

COX-2 protein levels were significantly decreased by 51.0% (*p* = 0.013) and 72.1% (*p* = 0.001) with AG-2X and AG-5X treatment, respectively, as compared with vehicle treatment. In the trend analysis, both IL-1β and COX-2 levels were dose-dependently decreased (*p* < 0.0001). Ethanol ingestion may activate the innate immune system release of pro-inflammatory cytokines such as IL-1β [14]. Previous findings also found increased levels of IL-1β in ethanol-induced gastric ulcer tissue [15]. IL-1β, a representative inflammatory cytokine with pleiotropic functions, plays a key role in the process of inflammation. Recent studies have found that IL-1β has adverse effects in severe mucosal inflammation [16]. Our study demonstrated that 28 days of AG supplementation markedly inhibited the production of IL-1β in ethanol-induced gastric ulcer and decreased gastric lesions. COX-2 is known to be involved in modulation of gastric mucosal integrity. The enhanced ulcerogenic response is mediated by COX-2 gene expression [17]. In the present study, pretreatment with 500 or 1250 mg/kg AG decreased COX-2 level as compared with vehicle treatment. Therefore, reduced COX-2 expression may be involved in the gastroprotective action of AG. AG supplementation may decrease the levels of inflammatory proteins in ethanol-induced gastric damage.

### 2.5. Effect of AG Supplementation on Ethanol-induced Expression of Apoptosis Proteins in Rat Stomachs

**Figure 5:**
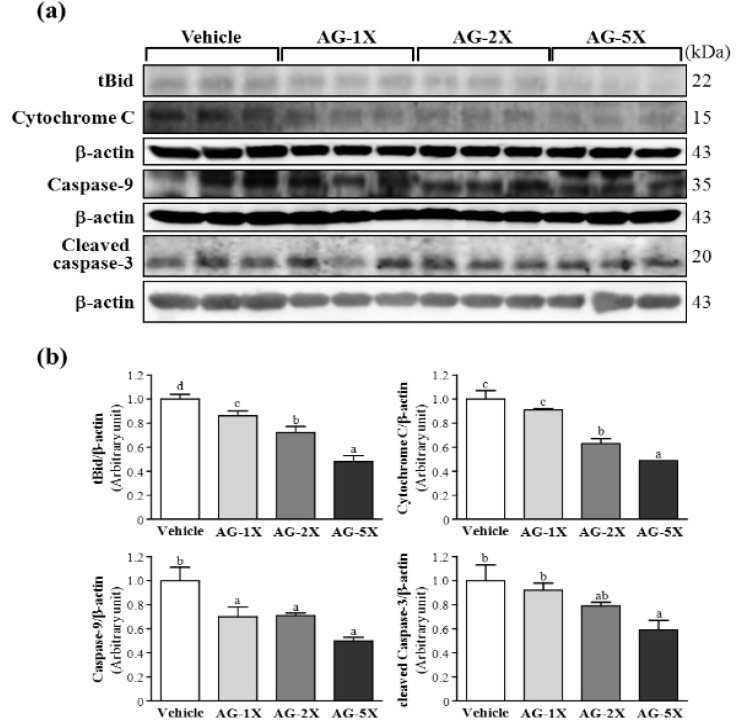
Effect of AG supplementation on ethanol-induced expression of apoptosis proteins in rat stomachs. Rats were treated as in Figure 4. (**a**) Western blot analysis and quantification of protein levels of tBid, cytochrome C, caspase-9 and cleaved caspase-3 in gastric tissues; and (**b**) Densitometry analysis was performed to quantify the expression levels of detected proteins. Data are mean ± SEM (n = 8 mice per group). Different letters (a, b, c, d) indicate a significant difference at *p* < 0.05.

Apoptosis is an important mechanism maintaining homeostasis during development and for response to external stimuli in multicellular organisms [18]. The mitochondrial pathway of apoptosis is mediated by the release of a number of factors from mitochondria. The release of cytochrome c is the central gate in turning on or off apoptosis and is regulated by the interaction of proapoptotic proteins, including caspase-3 and -9 [19]. tBid is initially found to be myristoylated and translocated into mitochondria in response to death-receptor–mediated apoptotic signaling [20]. We tested whether AG supplementation could protect against ethanol-induced protein expression of tBid, cytochrome C, caspase-9 and cleaved caspase-3. tBid protein levels were significantly decreased, by 14.3% (*p* = 0.0382), 28.2% (*p* = 0.0011) and 51.9% (*p* < 0.0001), with AG-1X, AG-2X and AG-5X treatment, respectively, as compared with vehicle treatment (Figure 5).

Cytochrome C protein levels were decreased by 37.0% (*p* = 0.003) and 51.5% (*p* < 0.001) with AG-2X and AG-5X treatment, respectively, as compared with vehicle treatment. Caspase-9 protein levels were significantly decreased by 29.5% (*p* = 0.0200), 28.8% (*p* = 0.0221) and 50.2% (*p* < 0.0001) with AG-1X, AG-2X and AG-5X treatment, respectively, as compared with vehicle treatment. Cleaved caspase-3 level was significantly decreased by 40.5% (*p* < 0.001) with AG-5X treatment as compared with vehicle treatment. In the trend analysis, tBid, cytochrome C, caspase-9 and truncated caspase-3 levels were dose-dependently decreased (*p* < 0.0001). A previous study reported that American ginseng could downregulate the pro-apoptotic factors caspase-9 and -3, thus reducing apoptosis [21,22]. The release of cytochrome C is the central gate turning on or off apoptosis and is regulated by the interaction of proapoptotic proteins Bid, Bax and Bak and anti-apoptotic proteins Bcl-2 and Bcl-xL. Therefore, AG supplementation may decrease pro-apoptosis protein levels and protect against ethanol-induced gastric injury.

### 2.6. Effect of AG Supplementation on Expression of Anti-Apoptosis Proteins in Rat Stomachs

**Figure 6:**
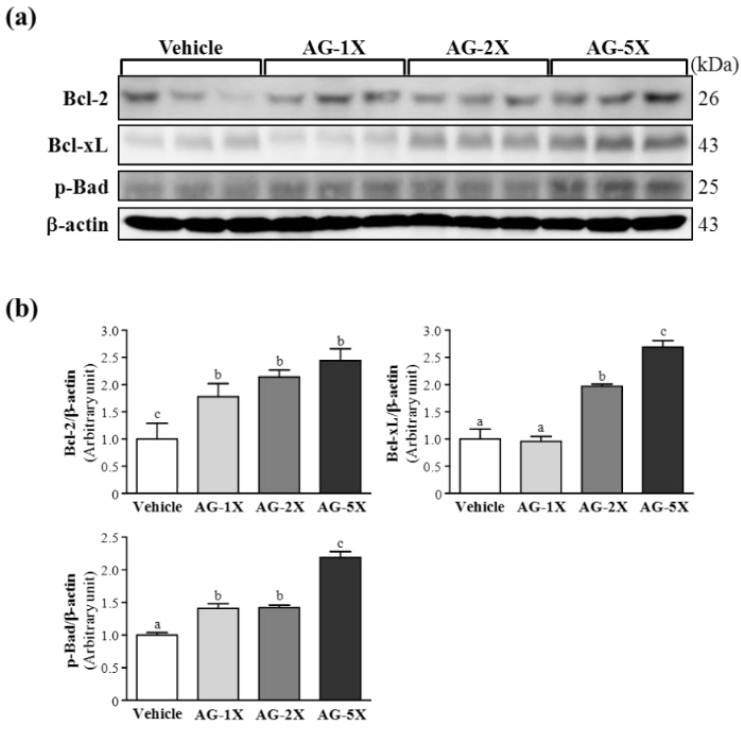
Effect of AG supplementation on anti-apoptosis protein expression in rat stomachs. Rats were treated as in Figure 4. (**a**) Western blot analysis and quantification of protein levels of Bcl-2, Bcl-xL and p-Bad in gastric tissue; and (**b**) Densitometry analysis was performed to quantify the expression levels of detected proteins. Data are mean ± SEM (n = 8 mice per group). Different letters (a, b, c) indicate a significant difference at *p* < 0.05.

The proto-oncogene Bcl-2 and its homolog Bcl-xL can inhibit apoptosis in biological systems [23]. Thus, we investigated whether AG supplementation has a protective role in ethanol-induced gastric damage through anti-apoptosis protein expression. Bcl-2 protein levels were significantly elevated by 1.78- (*p* = 0.0409), 2.14- (*p* = 0.0074) and 2.44-fold (*p* = 0.0020) with AG-1X, AG-2X and AG-5X treatment, respectively, as compared with vehicle treatment (Figure 6).

Bcl-xL protein levels were increased by 1.97- (*p* = 0.004) and 2.69-fold (*p* < 0.001) with AG-2X and AG-5X treatment, respectively, as compared with vehicle treatment. The expression of p-Bad was significantly increased by 1.41- (*p* = 0.017), 1.42- (*p* = 0.014) and 2.19-fold (*p* < 0.001) with AG-1X, AG-2X and AG-5X treatment, respectively, as compared with vehicle treatment. In the trend analysis, Bcl-2, Bcl-xL and p-Bad levels dose-dependently increased with AG treatment (*p* < 0.0001). Bcl-2 and Bcl-xL belong to the bcl-2-related gene family and act as broad anti-apoptotic factors extending both normal and tumor cell survival [24]. Bcl-2 and Bcl-xL can inhibit apoptotic death primarily by controlling the activation of caspase proteases [25]. Bcl-2 protein expression was elevated with ginseng extract administration to ameliorate apoptosis in gastric mucosa of patients with gastric ulcer [26]. In the present study, the expression of Bcl-2 was significantly increased in the gastric mucosa of all AG-treated groups. Similar results were observed for Bcl-xL protein expression. Overexpression of both Bcl-2 and Bcl-xL may downregulate ethanol-induced gastric mucosa-cell apoptosis. AG supplementation may increase the protein levels of anti-apoptosis factors and protect against ethanol-induced gastric injury.

## 3. Experimental

### 3.1. Chemicals and Antibodies

American ginseng extract was provided by Taiwan Biotech Co. (Taoyuan, Taiwan). All other chemicals were from Sigma-Aldrich Chemical Co. (St. Louis, MO, USA). We purchased primary antibodies against β-actin (Oncogene Science, Uniondale, NY, USA), tBid, Bcl-2 , Bcl-X_L_, p-Bad (Transduction Laboratories, Lexington, KY, USA), cytochrome C (BD Pharmingen, San Diego, CA, USA), cleaved caspase-3, caspase-9, COX-2, and IL-1β (Cell Signaling Technology, Beverly, MA, USA).

### 3.2. Induction of Gastric Ulcer and Histology

Specific pathogen-free (SPF) male Wistar rats (6 weeks old) were purchased from the BioLASCO (A Charles River Licensee, Yi-Lan, Taiwan). Standard chow and water were available *at libitum*. All animals were housed in the animal facility at Kaohsiung Medical University (KMU). Before experiments, rats were acclimated for 1 week to the environment and diet. The Institutional Animal Care and Use Committee of KMU approved all animal experimental protocols, and the study conformed to the guidelines of protocol IACUC-102016 approved by the IACUC ethics committee. 

In the first experiment, we investigated whether a chronic (28-day) supplementation with AG could protect gastric tissue against ethanol-induced ulcer. We randomly divided 32 male Wistar rats into four groups (n = 8 per group) for treatment: supplementation with water (vehicle) and low-dose (AG-1X), medium-dose (AG-2X) and high-dose (AG-5X) AG at 0, 250, 500 and 1250 mg/kg, respectively, for 4 weeks. At day 29, 75% ethanol was given orally to each animal at 10 mL/kg to induce gastric ulceration for 2 hr. Two hours after ethanol administration, all animals were killed and stomachs were removed, opened along the greater curvature and rinsed with saline to remove gastric contents and blood clots.

Gastric tissues were collected and fixed in 10% formalin after sacrifice. Then, each sample was embedded in paraffin and cut into 10-μm thick slices for morphological and pathological evaluation. Tissue sections were stained with hematoxylin and eosin (H&E) and examined under a light microscope as we previously described [27]. Each stomach was examined grossly and the degree of ulceration was graded as follows [28,29]: 0, no lesions (normal stomach); 0.5, hyperemia (red coloration); 1, hemorrhagic spots; 2, 1–5 small ulcers; 3, many small ulcers; 4, many small and large ulcers; 6, stomach full of ulcers with perforations.

### 3.3. Effect of AG Supplementation on Ethanol-Induced Gastric Mucosal Damage

We randomly divided 32 male Wistar rats into four groups (n = 8 rats per group) for treatment as described above. Rats fasted for 24 hr before the experiment but had free access to water. After 60 min, 70% ethanol was given orally to each animal at 10 mL/kg to induce gastric ulceration. At 4 h after ethanol administration, animals were killed with CO_2_ and stomachs were collected analysis.

### 3.4. Western Blot Analysis

Total protein of stomach tissues (0.1 g) from each rat was homogenized in a mixer ball mill (MM301, Retsch, Haan, Germany) for 2 min, extracted by adding 0.4 mL lysis buffer and centrifuged at 15,000 ×*g* for 30 min at 4 °C. Protein determination and western blot analysis were performed as previously described [30]. Protein content was measured by the Bradford method (Bio-Rad, Hercules, CA, USA). Proteins were resolved by 5%–20% gradient SDS-PAGE, then immunoblotted by use of enhanced chemiluminescence assay (ECL; Perkin Elmer Life Science, Boston, MA, USA) and images were obtained by use of ImageQuant LAS-4000. Quantification involved use of Alpha Ease FC (Alpha Innotech, San Leandro, CA, USA).

### 3.5. Statistical Analysis

All data are expressed as mean ± SEM. Statistical differences were analyzed by one-way ANOVA and the Cochran-Armitage test for dose-effect trend analysis with SAS 9.0 (SAS Inst., Cary, NC, USA). *p* < 0.05 was considered statistically significant.

## 4. Conclusions

American ginseng extract may be able to attenuate ethanol-induced gastric ulcers by modulating the inflammatory and apoptosis responses.
